# “O” no: a Reddit analysis of orgasmic dysfunction

**DOI:** 10.1093/sexmed/qfad061

**Published:** 2023-12-04

**Authors:** Rachael Belcher, Danielle Sim, Marcella Meykler, Jeunice Owens-Walton, Naeemul Hassan, Rachel S Rubin, Rena D Malik

**Affiliations:** Department of Surgery, University of Maryland Medical Center, Baltimore, Maryland 21201, United States; Division of Urology, Department of Surgery, University of Maryland School of Medicine, Baltimore, Maryland 21201, United States; School of Medicine, Albert Einstein College of Medicine, Bronx, New York 10461, United States; Division of Urology, Department of Surgery, University of Virginia School of Medicine, Charlottesville, VA 22903, United States; College of Information Studies, University of Maryland, College Park, Maryland, 20742, United States; Philip Merrill College of Journalism, University of Maryland, College Park, Maryland 20742, United States; Division of Urology, Department of Surgery, Georgetown University School of Medicine, Washington DC 20057, United States; Division of Urology, Department of Surgery, VA Long Beach Healthcare System, Newport Beach, California 92663, United States

**Keywords:** female orgasmic disorder, orgasm, climax, Reddit, social media

## Abstract

**Background:**

Female Reddit users frequently discussed potential causes of orgasm difficulties and its implications on mental health and relationships.

**Aim:**

This study aimed to evaluate the experiences of women discussing orgasms on the Internet site Reddit. We sought to qualitatively analyze the topics that arose in users’ discussions to better understand the potential causes of orgasm difficulties and its implications on quality of life.

**Methods:**

Posts on the subreddit r/TwoXChromosomes containing the keywords “orgasm” and “climax” were included in the dataset. Posts and their associated comments were qualitatively analyzed using the grounded theory approach. Two independent researchers coded each thread to identify dominant themes and emergent concepts.

**Outcomes:**

The most frequently coded primary topics included: (1) orgasm (32.2% [n = 337]), (2) psychological (17.8% [n = 186]), (3) relationships (15.4% [n = 161]), and (4) treatment (10.7% [n = 112]).

**Results:**

Qualitative analysis of 107 threads and approximately 6300 comments resulted in 5 major categories: psychological aspect of orgasms, difficulty orgasming with partners, partners’ responses to orgasmic dysfunction, types of orgasms, and treatments for orgasmic dysfunction. Preliminary themes included (1) the presence of an emotional component or history of trauma related to orgasmic difficulty, (2) difficulty orgasming with a partner regardless of ability to orgasm during masturbation and a variety of stimulation required to orgasm, (3) mixed partner responses to orgasmic dysfunction, (4) the definition of a normal orgasm, and (5) self-motivated treatment for orgasmic dysfunction, including clitoral stimulation devices and masturbation techniques. Notably, few posters discussed their orgasmic dysfunction with healthcare providers.

**Clinical Translation:**

The study reveals insights into the possible causes, psychosocial implications, and treatment of orgasm difficulties from a patient perspective, and can guide future research on female orgasms in a more precise, patient-oriented direction.

**Strengths and Limitations:**

The anonymous nature of the forum allowed for insight into sensitive topics related to female orgasms and sexual trauma. Limitations include the demographic distribution of Reddit users, which was primarily younger women in their 20s and 30s, which restricts generalizability.

**Conclusion:**

Reddit provides a medium for individuals with orgasm difficulties to discuss their experiences. Posts addressed users’ inability to orgasm, their mental health and relationships, the stimulation required for orgasm, and treatments for orgasmic dysfunction. Interestingly, very few posts discussed healthcare, potentially suggesting that women do not classify their orgasmic dysfunction as a health issue.

## Introduction

An orgasm is described as the sudden discharge of accumulated sexual excitement during the sexual response cycle resulting in circumvaginal musculature contraction and a sense of sexual pleasure.^1^ Orgasm achievement in both partnered and solo sexual activity can help contribute to women’s sexual health, which is considered a vital sign for overall health.^2^ Female orgasmic disorder (FOD) is characterized by a significant change in orgasm such as delay, reduction of intensity, or cessation.^3^ The prevalence of orgasmic dysfunction in the United States has been estimated at approximately 20.5%,^4^ making it one of the most common types of female sexual dysfunction. The scientific literature discussing orgasm difficulties reports potential explanations for female orgasmic dysfunction and the types of and differences between female orgasms.

A study performed by Rowland et al^5^ assessed attributes among women who reported difficulty reaching orgasm during partnered sex and found that the most frequently endorsed cause of inability to orgasm was stress or anxiety. Similarly, other studies concluded that higher cognitive distraction and sexual inhibition negatively predict orgasm frequency.^6^^,^^7^ A history of sexual trauma has also been associated with a reduced orgasm frequency.^6^

Other research has examined the modes of stimulation that women require to achieve orgasms and their perceptions of female orgasm during sex. When surveyed about the types of stimulation in relation to climax, some women endorsed that although they can experience orgasm through penetration alone, they do so infrequently.^8^ Women also confirmed that the likelihood of orgasm increased when clitoral stimulation was added.^8^ Additionally, many women believe their orgasm during sex is more important for their male partners than it is for themselves.^9^ As such, women are more likely to focus on their partner’s sexual pleasure and tend to avoid asking for additional stimulation due to fear of damaging their partner’s ego.^9^

Though the current literature outlines potential sources and complications of orgasm difficulties, these studies are limited by their research design, with the majority relying on medical surveys or limited patient interviews.^4-9^ This design allows for a more focused, hypothesis-driven data collection and analysis; however, it fails to account for the array of perceptions and experiences that women have regarding their sexual satisfaction and orgasm ability. Specifically, the current literature relies on the pathologizing of women’s sexuality, in that it presumes all women want and must enjoy sexual intercourse.^10^^,^^11^

Given that online medical websites, medical Web-based forums, and nonmedical social media sites are becoming a major source for individuals to discuss health-related information, we decided to study orgasm-related posts on an anonymous and public Internet platform.^12^^,^^13^ We intended to study women’s own descriptions of their sexual problems, rather than questions based on the ill-defined and contested concept of female orgasmic dysfunction, to reconceptualize women’s perceptions of sexual orgasm.

Former studies have failed to demonstrate how multiple psychosocial factors, such as pressure from sexual partners, sexual history, mental health, and self-image, influence individual perceptions about orgasms and orgasm dysfunction, and which of these factors are most frequently cited as a potential cause or consequence of women’s orgasm difficulties. Therefore, by analyzing anonymous online information from the Internet forum Reddit, we sought to gather a diverse collection of women’s perceptions and experiences with orgasms to provide a more comprehensive understanding of how women perceive orgasm-related challenges. Our research question is, “How are female Reddit users discussing their experiences with orgasms and what topics are commonly mentioned when users are sharing their accounts with orgasm difficulties?”

## Aim

This study aimed to evaluate the experiences of women discussing orgasm on the Internet site Reddit, an anonymous forum-based platform that allows users to interact with a global audience. We sought to qualitatively analyze the topics that arose in an anonymous, digital platform to better understand what elements of sexual climax are important to women as well as the potential causes of orgasm difficulties and its implications for quality of life. What topics relating to orgasm do female Reddit users frequently post about? Is the discussion surrounding female orgasm primarily focused on orgasm dysfunction, and if so, what do users site as the cause of their dysfunction and what aspect of this dysfunction do users find most distressing? Do the orgasm-related themes addressed by Reddit users mirror those studied in sexual medicine research and applied in sexual medicine practice?

## Methods

### Data source

The subreddit r/TwoXChromosomes was used for this study. Reddit is a prominent Web-based social media platform, with 52 million daily active users.^14^ Reddit users can interact with over 100 000 active communities called subreddits, or secondary threads that allow users to focus on a specific interest or topic.^14^ Within these subreddits, posted content and comments can receive and up vote or down vote based on user preference, which contributes to the order of posts within each thread. Due to its large user population, the precategorized subreddits based on topic, and its data accessibility, Reddit has recently become a popular tool among qualitative researchers in various fields.^15^

There are multiple characteristics of Reddit that influenced our decision to use it as a data source. The open-forum nature of the website allows for users to describe their experiences in their own words without limitation, creating greater opportunities to evaluate how women perceive their orgasm ability and frequency. Additionally, commenters often directly compare their challenges to the original poster’s experiences, which is not possible on anonymous surveys. We chose the specific subreddit r/TwoXChromosomes for 2 primary reasons. First, in our original data query, r/TwoXChromosomes was one of several subreddits with high activity related to the topic of orgasm. Second, r/TwoXChromosomes is promoted as having a predominantly female user population that discuss topics pertaining to women. The group’s self-made description is as follows:

“Welcome to TwoXChromosomes, a subreddit for both serious and silly content, and intended for women’s perspectives. We are a welcoming subreddit and support the rights of all genders. Posts are moderated for respect, equanimity, grace, and relevance.”

The demographic distribution of Reddit active users is reported to 56% male.^14^ Therefore, a women-centered subreddit was thought to be advantageous for the purposes of this study, as users interacting with a subreddit community with gender-specific topics are more likely to be of said gender and to disclose their gender.

Of note, r/TwoXChromosomes is a moderated subreddit. Therefore, threads posted to the site are expected to follow the guidelines defined on the subreddit’s homepage. Posts that violate these guidelines are subject to removal by the moderator, which has the potential to influence the content of analyzed posts. A more detailed description of the subreddit rules and moderator approved topics can be found in Appendix A.

### Data collection

Data collection was completed using the Reddit’s application programming interface (API), a publicly available software that allows users to mine data from Reddit forums using keywords. This extracted data can then be externally manipulated and analyzed for research. Using Reddit’s API, we extracted posts that included the keywords “orgasm” and “climax.” The keywords were chosen based on a popular terms search on Google from the time of the subreddit’s creation to the time of data collection. Although the term “coming” was also frequently searched, due to its colloquial use in the English language we opted to exclude it as a keyword to prevent the collection of unrelated Reddit threads.

From the subreddit’s creation in 2009 until the point of data collection in November 2020, a total of 107 threads were analyzed, with 23 of the threads queried from the keyword “orgasm” and 84 from the keyword “climax.” Posts that were deleted or unrelated to orgasm/climax were excluded from our analysis. Of the analyzed threads, posting dates ranged from June 2016 to November 2020. In addition to the original forum threads, the analysis included all 6338 comments responding to the posts.

Posts on r/TwoXChromosomes that included the keywords were mined from Reddit’s public API and organized in Python (version 3.8.6; Python Software Foundation). Posts were then transferred and manually coded in Microsoft Word (version 16.69) and converted to xlsx format using Spyder (version 4.1.5; Spyder Doc Contributors) on Anaconda.Navigator (version 1.10.0; Anaconda Inc). Phrases and their associated codes were analyzed using Microsoft Excel (Excel 365; version 16.77.1).

Given the public nature of Reddit data, the University of Maryland Medical Center Institutional Review Board exempted this research from review.

### Content analysis

Threads were qualitatively analyzed using the grounded theory approach, a research technique that generates a theory grounded in data that has been systematically collected and analayzed.^16-18^ Posts including the keywords “orgasm” and “climax” were selected for analysis. Data analysis of the posts was carried out according to the steps described by Strauss and Corbin.^18^ The researchers began by coding each line of the original post along with the associated comments. Each phrase could be linked to a singular code. Similar codes were then grouped together to form subcategories. The major categories were then determined from the subcategories and used to form a theory related to female Reddit users’ experiences with orgasm. The codebook was updated throughout the coding process by the researchers until thematic saturation was reached. The final codebook contained a tripartite structure, with the major categories based on the subcategories, which were generated from the initial codes.

To measure consistency between coders, ensure the quality of coding, and limit systematic error and bias, interrater reliability was calculated using the formula described by Miles and Huberman^19^:


$$ reliability=\frac{number\ of\ agreements}{number\ of\ agreements+ disagreements} $$

Ten (9.3%) posts and their associated comments were analyzed for interrater reliability. Each coder analyzed the same 10 threads that were randomly sampled from the keyword “climax,” with the number of comments ranging from 3 to 290. Excerpts and their associated codes were analyzed for agreements and disagreements between pairs, with reliability equal to the number of agreements divided by the sum of agreements and disagreements. The goal of >80% reliability was achieved for the coders. The high consistency between coders ensures that the coding frame, or the relationship between main categories, subcategories, and codes, is reliable. In other words, although a code could fit in more than 1 category, the coders agree on the thematic structure of the categories.^20^

When coding was complete, a single researcher determined the overall frequency of a given category and the frequency of codes within each category. Initial analysis of the data showed that the number of codable phrases from threads with greater than 200 comments and those with 50 or fewer comments was similar, with an average of 33 phrases for the former and 21 phrases for the latter. This was due to the large number of comments providing reassurance or support without contributing additional information about female orgasms. Given these results, the researcher team made the decision to include the codes from the comment sections into the overall code count to better assess which topics were more popular among female Reddit users.

Of the 107 threads analyzed, 24.3% (n = 26) of posters disclosed their age and 17.8% (n = 18) stated their sexuality. The majority of these self-identifying posters were heterosexual women in their 20s and 30s (Appendix B).

## Results

Codebook categories included: (1) post characteristics, (2) labels/sexuality, (3) sexual dysfunction, (4) libido, (5) orgasm, (6) masturbation, (7) types of sex, (8) psychological factors, (9) relationships, (10) intimacy, (11) treatment, (12) medications, (13) healthcare, (14) questions, (15) suggestions, and (16) resources. In the final analysis, frequencies of orgasm-related codes were calculated with the exclusion of certain residual categories, namely post characteristics, labels/sexuality, questions, suggestions, and resources, to better analyze the primary topics related to female orgasm. Excluding these categories, the most frequent topics included: (1) orgasm (32.2% [n = 337), (2) psychological (17.8% [n = 186]), (3) relationships (15.4% [n = 161]), and (4) treatment (10.7% [n = 112]) (see Table 1).

**Table 1:** Category frequency and associated top 3 common codes in descending order of frequency.

**Category**	**Data**	**Common Codes**
Orgasm	32.25 (337)	Difficulty orgasming with partner Mental block to orgasm Unable to have vaginal orgasm
Psychological	17.80 (186)	History of trauma Feeling abnormal Societal pressures
Relationships	15.41 (161)	Partner interested in my sexual pleasure Supportive sexual partner Partner not interested in my sexual pleasure
Treatment	10.72 (112)	Clitoral stimulation device Sex toys Pelvic floor physical therapy
Masturbation	7.56 (79)	Unable to masturbate with hands Length of time with masturbation Lack of arousal with masturbation
Sexual dysfunction	7.08 (74)	Sensitivity issues Vaginismus Pain with clitoral stimulation
Type of sex	5.26 (55)	Sex for enjoyment without orgasm Oral sex Vaginal intercourse
Medications	1.53 (16)	Anorgasmia secondary to antidepressants Medication induced sexual dysfunction Decreased libido secondary to antidepressants
Intimacy	1.34 (14)	Trust required for intimacy Sex as intimacy Intimacy more important than sex
Healthcare	0.86 (9)	Dissatisfied with HCP Plan to talk with HCP Delayed diagnosis of sexual dysfunction
Libido	0.19 (2)	Decreased libido Increased libido

Values are % (n).

Abbreviation: HCP, healthcare provider.

### Orgasm

The conversation around orgasm primarily centered on a few reoccurring themes (see Table 2), with the most common being “difficulty orgasming with partner” (12.2% [n = 40]). Users reported an inability to orgasm with sexual partners, regardless of the length of the relationship or the ability to orgasm from masturbation. The theme of “mental block to orgasm” was often coded in conjunction with difficulty orgasming with a partner, accounting for 10.9% (n = 36) of orgasm-related codes. Many posters explained that a feeling of anxiety or pressure during partnered sex prevented them from reaching climax, with one user stating, “When I’m with a new sex partner, I rarely climax. It’s difficult for me to let down the barriers enough. Sometimes (e.g., when I’m particularly stressed), I have a very difficult time climaxing (alone or with my partner).” Other users described being able to overcome this mental block to orgasm in sexual situations where they felt more relaxed, had greater trust in their partner, or felt comfortable with their own sexual responses. As a result, these women reported greater orgasm frequency and labeled their sexual relationships with more positive identifiers. However, users acknowledged that feeling comfortable enough to climax with a partner was an involved and difficult process.

**Table 2:** Orgasm codes with the highest frequency and representative quotes.

**Code**	**Data**	**Representative quote**
Difficulty orgasming with partner	12.16 (40)	“I’ve been with my current partner for 3 years and everything is great, but I’ve reached a point now where I’m sick of feeling frustrated and ‘broken’ because I can’t orgasm with him.”
Mental block to orgasm	10.94 (36)	“I get anxious and scared when I have sex with others and because of that I can’t climax.”
Unable to orgasm from vaginal stimulation	10.33 (34)	“I actually find it really hard to cum, and rarely do, with just penetration, no matter how much foreplay I’ve had beforehand.”
Relaxation or trust required for orgasm	9.42 (31)	“When I’m with a new sex partner, I rarely climax. It’s difficult for me to let down the barriers enough.”
Vaginal orgasm	8.51 (28)	“I had my first ever orgasm from penetration and I still can’t believe that this has happened.”
Anorgasmia	8.21 (27)	“I’ve tried a variety of toys and lubes and even the old shower-head trick and I just can’t seem to find the thing that suits me.”
Clitoral orgasm	6.69 (22)	“Penetration doesn’t work for me and never has. I need direct clitoral stimulation and that’s normal.”

Values are % (n).

Additional regularly addressed topics included the stimulation required for orgasm and different types of orgasm. Threads often discussed the difficulty of orgasming from vaginal stimulation alone and the need for clitoral co-stimulation (10.3% [n = 34]). Notably, there was a significant discussion around vaginal orgasms (8.5% [n = 28]). A subset of users labeled vaginal orgasms as the ultimate fulfilling outcome of penetrative sex and their corresponding feelings of shame when unable to climax from that stimulus. When discussing her inability to orgasm via vaginal penetration, one poster expressed, “Thank you for writing this. I always felt inadequate as a woman, like there was something wrong with me because I can’t vaginally orgasm. I felt guilty and my husband at times felt the same way. All women are built differently and their bodies orgasm differently. This being whether it is clitoral or not. I feel so much better running across this post thread today.”

Other posters argued against society’s push for vaginal orgasms and advocated for general sexual enjoyment through various sexual stimuli, including clitoral stimulation. Regarding the various methods of sexual fulfillment, one user said, “It needs to be said, not everyone climaxes the same way. Not everyone climaxes easily, or at all. It doesn’t mean you can’t take pleasure in sex, and absolutely doesn’t mean you’re not a ‘real’ woman.” Another user advocated for clitoral stimulation in sexual encounters by saying, “Expecting a woman to come without anything touching her clit is like expecting a guy to come without anything touching his penis. It can happen for some people but is impossible for others.”

Of the codes pertaining to orgasm, 5 (1.5%) codes described orgasming too quickly, or premature orgasm. Examples of phrases linked to premature orgasm include, “Once I’m stimulated down there (‘plateau phase’), I can barely last 2 minutes, with the really pleasurable part only lasting like 30 seconds, unless I go super slow or practice edging (and still have to go slowly), which I feel just kills the fun” and “I don’t think that I’ve ever met anyone as quick as I am, but I usually climax within a couple minutes when I’m by myself.”

There were 4 coded phrases that mentioned female ejaculation. Of the 4, 3 of linked phrases discussed female ejaculation in pornography, rather than the user’s personal experience.

### Relationships

Topics pertaining to relationships were recurrently mentioned alongside discussions on orgasmic dysfunction. When discussing their difficulties orgasming during sex, users often stratified their sexual partners as “interested in my sexual pleasure” and “not interested in my sexual pleasure.” Partners who were labeled as interested in the posters’ sexual pleasure were described as patient and willing to learn about the poster’s desired sexual stimuli. Women who expressed positive sexual relationships often reported greater partner communication, resulting in less anxiety during sexual encounters. One user, who was comparing her previous relationships with her current sexual partner, said, “Now, years later, I’m seeing a man who is actually the first person to suggest using the toy. I asked him today if he felt weird about it, and he just smiled and said ‘No, I just really like seeing you feel good. Thank you for letting me be part of that.’ I was a little blown away by that I guess.”

Conversely, partners who were labeled as uninterested in the posters’ sexual pleasure were described as selfish and insecure, with one user stating, “There are no conversations about my orgasm unless it’s ‘did you come?’ Or ‘how was it?’” Posters with this type of partner revealed long-standing sexual complications in their relationships. There were often corresponding feelings of insecurity, best summarized by one user’s comment: “I feel obsessive and stupid … for making it a ‘big deal’ but when every partner you’ve had, whether long or short term, completely dismisses your needs, feelings, and wants, you feel like an obligation.”

The majority of the relationship codes were classified as positive or neutral (52.5% [n = 83]). Related excerpts explained constructive communication between partners, partners’ understanding of posters’ orgasmic dysfunction, and a willingness to satisfy the posters’ sexual needs. The relationship codes that were classified as negative (47.5% [n = 75]) discussed partners’ dissatisfaction with the posters’ orgasmic dysfunction, partners’ feelings of threatened masculinity secondary to a lack of female orgasm, and posters’ fear of their partners’ reactions to their sexual issues.

### Psychological factors

When examining how orgasm interacts with various psychological factors, our analysis revealed trauma as a prominent theme (19.4% [n = 36]). Previous sexual trauma was reported to continuously affect a woman’s ability to orgasm and enjoy sexual stimulation, which led to women feeling “abnormal” or “broken” (19.4% [n = 36]). History of trauma was often coded alongside the previously mentioned themes of “difficulty orgasming with partner” and “mental block to orgasm.”

**Figure 1:**
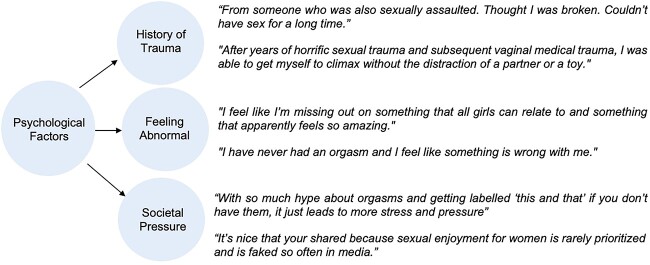
Example user quotes for frequently coded psychological factors related to orgasm difficulties.

Users discussed the societal pressure and stereotypes that surround female orgasms, citing the expectation of orgasm during intercourse and the propensity of the media to exaggerate female sexual pleasure and climax (see Figure 1). Pornographic stereotypes of sexual acts and the expected female response to sexual pleasure were mentioned in response to posters describing their orgasms as “abnormal.” Other common psychological codes concern women’s feelings of distress surrounding their sexual dysfunction. Codes of guilt, shame, distress, feelings of inadequacy, fear, anxiety, desperation, depression, and stress accounted for 20.4% (n = 38) of all psychological codes. When discussing her challenges with climaxing, one user stated, “I feel selfish and greedy wanting someone to take time to make me feel good.” Another user voiced similar feelings, stating, “I hate to feel ashamed for something I have absolutely no control over. I hate to feel sorry for my boyfriend, like I owe him an apology, because I still don’t have an orgasm with him.” Codes pertaining to positive emotions included relief, prioritizing sexual function, feeling empowered, and accepting oneself (14.5% [n = 27]).

### Treatment options and healthcare

Users frequently discussed treatments for difficulty orgasming with or without a partner. Posts related to clitoral stimulation devices (CSDs) accounted for 37% (n = 41) of the discussions on treatment options. Users mentioned the benefits of CSDs when exploring their ability to climax through masturbation and during partnered sex. Similarly, commenters often cited other types of sex toys as a potential solution for orgasm difficulties. Other common treatment-related codes included pelvic floor physical therapy, counseling, pornography, and supplements (see Table 3).

**Table 3:** Codes pertaining to treatment and relative frequency (n = 112).

Clitoral stimulation device	36.61 (41)
Sex toys	33.04 (37)
Pelvic floor physical therapy	8.93 (10)
Therapy or counseling	8.04 (9)
Pornography	7.14 (8)
Supplements	1.79 (2)
“Spicing it up”	0.89 (1)
Cannabis	0.89 (1)
Failed self-treatment	0.89 (1)
Scheduling sex	0.89 (1)
Lubricants	0.89 (1)

Values are % (n).

Interestingly, users rarely mentioned healthcare in the context of orgasmic dysfunction (0.9% [n = 9]). Dissatisfaction with healthcare providers, frustration with the healthcare system, and delayed diagnosis of sexual dysfunction accounted for 55.6% (n = 5) of healthcare-related codes. Three posters reported a plan to discuss their sexual dysfunction with a healthcare provider.

### Additional categories

The infrequently coded categories included masturbation, sexual dysfunction, types of sex, medications, intimacy, and libido. Codes related to masturbation encompassed 7.6% (n = 79) of the total codes, with users discussing the inability to masturbate without sex toys, the length of time required for masturbation, and the inability to feel aroused during masturbation. The sexual dysfunction category encompassed 7.1% (n = 74) of the total codes, with most of its discussion surrounding sensitivity issues, vaginismus, and pain with clitoral stimulation. The types of sex category (5.3% [n = 55]) dissected the role of orgasm in sex and whether climax was necessary for sexual enjoyment, oral stimulation, and vaginal intercourse. Users infrequently discussed medications (1.5% [n = 16]), but within that category the most frequent codes addressed anorgasmia or other sexual dysfunction secondary to medications, particularly antidepressants. The intimacy category (1.3% [n = 14]) included codes such as “trust required for intimacy,” “sex as intimacy,” and “intimacy more important than sex.” Finally, codes related to libido (0.2% [n = 2]) addressed the decrease or increase in users’ baseline libido levels.

## Discussion

Our study revealed that a large population of female Reddit users in this thread were interested in discussing topics pertaining to orgasms.

### Orgasm difficulties

The most frequently reported challenge was difficulty orgasming with sexual partners. Users’ inability to orgasm during partnered sex did not correlate with general anorgasmia, as many posters reported regular orgasm frequency during masturbation. The explanation unveiled a mental block that prevented these women from enjoying sex freely enough to achieve orgasm. Similar findings previously reported in the existing literature. The prevalence of FOD is estimated to be approximately 20% in sexually active women.^4^ Failure to orgasm during partnered sexual activity has been reported in 49.5% of women.^5^ Many women, both in our Reddit analysis and in the existing literature, cite their anxiety as the main cause of difficulty climaxing during sex. Rowland et al^5^ examined attributions and thought processes in a population of women with difficulty reaching orgasm and found that stress or anxiety during sex was correlated with greater difficultly orgasming, lack of arousal during sex, and negative body image. Tavares et al^7^ found that the threats of performance failure and performance consequences negatively affected the frequency of orgasm, indicating that the presence of sexual inhibition is potentially more relevant than the absence of sexual excitation for female orgasm. Other studies have found that high levels of cognitive distraction during sexual activity led to lower self-esteem, decreased sexual satisfaction, fewer orgasms, and a greater propensity to fake orgasms.^21^ Our study underscored these findings, as many female Reddit users described a mental block preventing them from reaching climax, regardless of their desire during sexual activities. Reddit users also shared their experiences with their partners’ disappointment secondary to orgasmic dysfunction. Notably, women who expressed positive relationship status often simultaneously voiced less anxiety and greater communication with their sexual partners. These findings reveal a potential negative loop inhibiting a woman’s ability to climax; women who have anxiety surrounding their orgasm ability will subsequently worry about their partner’s satisfaction, further adding to the pressure to reach climax. If this negative loop occurs repetitively in sexual interactions, it can lead to distress and decreased quality of life.

### Sexual stimuli

In addition to discussing the cognitive elements required for orgasm, users debated the types of sexual stimuli and their ability to orgasm during said stimuli. Prior research has demonstrated that, although women are able to orgasm solely through vaginal penetration, the frequency and enjoyment of orgasms increases with the addition of clitoral stimulation.^8^ While Reddit users endorsed this concept, many posters expressed a desire for orgasm from heterosexual penetrative sex without clitoral stimulation. The desire for vaginal orgasms, or what users refer to as PIV (penis-in-vagina) orgasms, may stem from the heteronormative societal expectations of partnered sex. Lavie-Ajayi and Joffee^22^ examined how societal expectations of female orgasms influence women’s beliefs. They found that women tend to view clitoral orgasms as achieved via self-stimulation and vaginal orgasms as achieved through sex with a man. Furthermore, responders described the latter as “the real thing,” “better,” “deeper,” and “a sign of real womanhood.”^22^ In the current study’s threads discussing vaginal orgasms, a few users attempted to counteract previous posts labeling vaginal orgasms as superior by describing the female orgasm seen in pornography as exaggerated and unrealistic, based on the stimuli received. However, regardless of any counterarguments, the original posters still voiced their desire for vaginal orgasms, underscoring the idea that societal expectations and ideology can influence women’s beliefs about their sexual function.

### Psychological factors

Psychological factors influencing orgasm have also been addressed by Reddit users, and prior sexual trauma has emerged as a main cause of orgasmic dysfunction. Sexual abuse and trauma in childhood have previously been associated with decreased desire and orgasm difficulties.^6^^,^^23^ Therefore, the high frequency of posts related to sexual trauma among Reddit users discussing orgasmic dysfunction is fitting given the current understanding of the negative impact of sexual trauma on global gynecologic and sexual health.

The DSM-5 requires the criterion of personal distress secondary to sexual dysfunction for the diagnosis of any type of female sexual dysfunction, including FOD.^3^ Of the women who report difficultly reaching orgasm during partnered sex, 58% reported distress about it.^5^ Additionally, prior studies have associated difficulties in reaching orgasm with self-blame, control, repressed emotions, greater dependency, apprehensiveness, and negativity.^7^ The population of Reddit users assessed in this study demonstrated high levels of distress and similar negative emotions, secondary to their sexual dysfunction. It is unclear whether these women would meet the criteria for FOD, as it is difficult to separate personal distress from relationship distress, especially given that women commonly address orgasmic dysfunction in the setting of partnered sexual activity.

Furthermore, there was a subset of Reddit users who reported inability to orgasm during sex without associated feelings of distress or anxiety, a finding that relates to the pathologizing of female sexual function. This sexual medicine topic expands on the history of female sexual dysfunction classification and its basis on the male model of sexual health, in that all women should have the same sexual drive and sexual response.^10^^,^^11^ The tendency to diagnose and categorize variations in women’s sexual response has been debated and critiqued by various sexual medicine researchers.^10^^,^^11^

Therefore, our findings on ambiguity surrounding the type of distress and the variation in women’s feelings about their ability to orgasm may indicate the need for re-evaluation of the current diagnostic criteria and expansion of what is considered normal female sexual function.

### Treatment options

Of the treatment options discussed by Reddit users, CSDs were most frequently mentioned in orgasm-related threads. Conversations around CSDs examined their use in both partnered sex and masturbation. Some users discussed the need for CSD in sexual activity because of an inability to climax from manual clitoral stimulation with their or their partners’ hands. Other users expressed their inability to experience an orgasm with any stimulation, including CSDs. Reddit posters rarely discussed their partners’ reactions to CSDs. This contradicts the findings of the previously mentioned study examining gender beliefs of the female orgasm, in which women expressed fear of their partners’ reactions if they requested clitoral stimulation, either manually or through CSD.^9^ In the same study, men stated that they were not offended when women asked for manual stimulation but expressed indifference or negative feelings toward using a vibrator to stimulate the clitoris.^9^ Given the recent societal push for female sexual enjoyment and sexual exploration, further research is needed to examine the current gender beliefs of CSDs and other sex toys.

### Healthcare

As previously stated, few female Reddit users mentioned healthcare in their discussions on orgasmic dysfunction. The shortage of users seeking medical counseling for orgasm challenges suggests that women do not classify their orgasmic dysfunction as a health issue and, therefore, do not seek care. There are various potential reasons why women do not consider orgasmic dysfunction to be a medical issue. First, women who are unable to orgasm during partnered sex yet can do so through masturbation are confident in their physiologic ability to climax. In these cases, women may attribute decreased orgasm frequency to inadequate communication in their relationships or their own beliefs about the purpose of sex. Other studies have demonstrated that many women view orgasming during sex as a bonus, rather than the primary goal.^9^ Another explanation for the lack of healthcare involvement in orgasm-related sexual health is that many women can attribute their sexual dysfunction to a source, namely, sexual trauma. In this case, women may not see the role of healthcare providers in counseling them on this issue or may not want to disclose their abuse history to their provider. Sexual assault prevalence data suggest that although 20% to 28% of women have experienced sexual assault,^24^^,^^25^ only 2% to 16% of these women have disclosed the assault to a physician.^24^^,^^26-29^

However, the large number of women who are distressed about their sexual functioning and seek counseling online indicates that they view their sexual responses as abnormal. Therefore, seeking medical counseling for their symptoms would be appropriate, yet many women fail to do so. Prior studies have shown that many women are reluctant to initiate discussions about their sexual health concerns. Only 35% of women reported female sexual dysfunction of their own volition, vs the 69% of women who reported dysfunction when asked directly.^30^ In one survey, 71% of adults said they thought their doctor would dismiss any concerns about sexual problems they might introduce, and 68% said they were afraid that discussing sexuality would embarrass their physician.^31^ Similarly, among Reddit users who talked to providers about their FOD, the majority reported negative experiences.

Patients rely on their providers to start an open and safe dialogue on sexual health.^32^ Unfortunately, there are low rates of female sexual dysfunction screening practices among providers, and many physicians feel inadequately trained to address such issue.^33^ Fear of offending patients, the possibility of patient denial, and concern for potential perceived sexual harassment were expressed as reasons for deferring screening. The low rate of patients seeking medical care for orgasmic dysfunction, the limited FOD knowledge among and screening by healthcare providers, and low rates of patient satisfaction among those who do discuss it with a provider all reveal a scarcity of adequate sexual dysfunction healthcare that negatively affects diagnosis and treatment of orgasm disorders.

### Strengths and limitations

The current study has several limitations. First, the cross-sectional design of the study limits its generalizability, as the results are only representative of the moment in time that the data were collected. Similarly, data mining from a singular subreddit and the inclusion of only 2 search terms may constrain the topics and perspectives of Reddit users. Additionally, because the study analyzed qualitative data, the self-reported symptoms, experiences, and demographics of Reddit users could not be verified. Moreover, the results may have limited generalizability to the greater population, as the data was queried from one type of social media platform and has a younger demographic distribution, which excludes issues prevalent among middle-aged and elderly women such as vaginal dryness and vulvovaginal atrophy.^34^ Similarly, the article does not consider that a portion of the Reddit posters may be on the asexuality spectrum and working from a perspective of internalized allosexual beliefs.^35^^,^^36^ Finally, prior studies caution researchers on using Reddit as a data source, as the structure relies on a specific Internet culture that influences which conversations are most visible, which limits the overall accuracy and applicability of Reddit-sourced data.^11^^,^^37^ Future studies should expand on these results, including additional social media platforms and ensuring data is collected from a more diverse population.

Despite its limitations, the current study’s design allowed for greater insight into sensitive topics related to female orgasms and orgasmic dysfunction. Reddit’s unique forum format creates the opportunity for women to cite their unique experiences and use their own terminology when describing their orgasmic functioning. This anonymous, unregulated design allows for a renewed analysis of women’s sexual problems without the preconceived notions of sexual medicine or fear of judgment. These results can guide further investigations into the potential causes of orgasmic dysfunction, including personal attributes that may decrease orgasm frequency. Additionally, our study demonstrates that some female Reddit users attribute their history of sexual trauma to their current sexual dysfunction, indicating the need for more comprehensive FOD and sexual trauma screening by healthcare providers. Finally, variation in women’s perceptions of their orgasm ability, as well as the interplay of orgasm difficulties with relationships and mental health, suggests that the current female orgasmic dysfunction diagnostic criteria should be re-evaluated to avoid pathologizing female sexual health.

## Conclusions

Our study resulted in 5 major female orgasm-related themes reported by Reddit users: (1) history trauma effecting orgasm ability, (2) difficulty orgasming with a partner regardless of ability to orgasm during masturbation and a variety of stimulation required to orgasm, (3) mixed partner responses to orgasmic dysfunction, (4) a tendency to label orgasms normal or abnormal, and (5) self-motivated treatment for orgasmic dysfunction, including clitoral stimulation devices. Notably, a few posters discussed orgasmic dysfunction with healthcare providers. Overall, the current study reveals that challenges with female orgasm are heavily influenced by trauma and social perceptions of normal sexual functioning, and that this difficultly orgasming is most often experienced in partnered sexual activity, rather than masturbation. These results can be used as a tool to guide future research on female orgasms in a more precise direction.

## Supplementary Material

fod_appendix_a_qfad061

appendix_b-fod_qfad061
