# Supplementary material for: “O” no: a Reddit analysis of orgasmic dysfunction
Source: Sex Med. 2023 Dec 4;11(6):qfad061. doi: 10.1093/sexmed/qfad061 (PMC10695429; doi:10.1093/sexmed/qfad061)
Supplement: fod_appendix_a_qfad061 [file fod_appendix_a_qfad061.docx]

**The 2xc community is encouraged to follow the general reddit guidelines, also known as the redditquette. Additional rules specific to the 2xc reddit are listed below.**

**Respect**

No hatred, bigotry, assholery, utter idiocy, misogyny, misandry, transphobia, homophobia, or otherwise disrespectful commentary. Use reddiquette. Please note that this includes behavior toward moderators. While we welcome constructive feedback and do our best to be responsive, we may not always be able to answer to your liking. We see no reason to require fellow mods to endure attacks, disrespect, or shitty behavior.

**Equanimity**

No drama-inducing crossposting of content found in other subreddits. Likewise, posts found to direct odious influxes here will be removed. Advertising your fabulous new subreddit is always welcome here.

You are welcome to link to relevant subreddits when warranted but linking to comment threads is almost always considered drama-inducing regardless of intent. Even with the best of intentions large groups following a link to a comment thread skew voting and commenting in a way that may be antithetical to the community being linked. If the comment thread you wish to linked to is already archived exceptions may be made.

Relevant AMA's in subreddits with over 500k subscribers may be advertised on the day of the post by anyone if a moderator of that subreddit requests removal we will be happy to comply. Smaller subreddits hosting AMA's must be posted by a moderator of that subreddit on the day of the AMA. Please give us 2-3 days notice through our modmail.

**Grace**

No tactless posts generalising gender. We are a welcoming community. Rights of all genders are supported here.

**Relevance**

We ask that you keep this community awesome by submitting content that is relevant to our experiences as women, for women, or about women. The following posts are generally not considered relevant:

- Medical Advice
- Relationship repair and advice about cheating
- Surveys, Research and Petitions
- Rage comics, memes, and motivationals
- Horrible things said and done by private individuals
- Any fundraising posts
- Posts about issues affecting men too
- Questions asking for the perspectives of women
- Posts made to debate the women of reddit
- Images outside of image guidelines
- Asking others to PM/chat you or offering to PM/chat with others
- Fundraising

Appendix A. Rules of the subreddit r/TwoXChromosomes.
