# Supplementary material for: “O” no: a Reddit analysis of orgasmic dysfunction
Source: Sex Med. 2023 Dec 4;11(6):qfad061. doi: 10.1093/sexmed/qfad061 (PMC10695429; doi:10.1093/sexmed/qfad061)
Supplement: appendix_b-fod_qfad061 [file appendix_b-fod_qfad061.docx]

| Poster Characteristics | N (% of Total Posts) |
| --- | --- |
| Age |  |
| <20 | 1 (0.9%) |
| 20s | 22 (20.6%) |
| 30s | 3 (2.8%) |
| Sexuality |  |
| Heterosexual | 13 (12.2%) |
| Homosexual | 2 (1.9%) |
| Asexual | 3 (2.8%) |
| Questioning | 1 (0.9%) |

Appendix B. Demographic distribution of Reddit posts.
